# Evaluation of insulin-like growth factor-1 in apparently healthy infants and prepubertal Egyptian children with different nutritional statuses

**DOI:** 10.1186/s12887-024-05118-x

**Published:** 2024-10-22

**Authors:** Hanan Mina Fouad, Amal Ahmed Mohamed, Nashwa Adel, Mohamed Abdulhay, Iman Khalifa, Randa Ibrahim, Naglaa Elsalway, Ghada Maher Thabet, Karima Nasraldin, Ingy Maher El-Hefny, Marwa S. Abd El-raouf, Dalia Ghareeb

**Affiliations:** 1 Pediatrics department, Faculty of Medicine, Helwan University, Cairo, Egypt; 2 Department of Biochemistry & Molecular Biology, National Hepatology & Tropical Medicine Research institute, GOTHI, Cairo, Egypt; 3 Clinical and Chemical Pathology Department, National Nutrition institute GOTHI, Cairo, Egypt; 4 Department of Clinical and Chemical pathology, Faculty of Medicine, Cairo University, Cairo, Egypt; 5 Faculty of Biotechnology, Modern Sciences and Arts University, Giza, Egypt; 6 Department of Public Health, Faculty of Medicine, Benha University, Benha, Egypt; 7 Department of Clinical Pathology, Faculty of Medicine, Suez University, P.O. Box 43221, Suez, Egypt

**Keywords:** Anthropometric measures, Breastfeeding, Gender, IGF-1, Obesity, Short stature

## Abstract

**Objectives:**

to estimate insulin-like growth factor-1 (IGF-1) levels in apparently healthy infants and prepubertal children and compare results among different nutritional statuses.

**Methods:**

Our cross-sectional work is a sub-study of a screening project for anemia and nutritional status. We included 252 apparently healthy infants and children with a mean age of 3.7 ± 1.3 years (1.1–6.6), with equal gender distribution. Data retrieved included breastfeeding and anthropometric measures. We tested the stored blood samples for IGF-1 levels. The sample size was reached when all kits were consumed.

**Results:**

abnormal anthropometric measures were detected in 32.9%, either a single or multiple, and 86.5% were breastfed. Girls had significantly higher serum IGF-1 levels than boys (P: <0.001), which was noticeable in girls with abnormal nutritional status detected with anthropometry. Breastfeeding showed no significant association with IGF-1 levels. No significant difference was observed between IGF-1 levels between children with normal versus those with abnormal growth measures. Children with overweight or obesity had significantly lower IGF-1 than children with other body mass index (BMI) categories. Serum IGF-1 levels correlated positively with arm muscle area Z scores in infants and toddlers and weight and BMI Z scores in children between three and four. Also, IGF-1 correlated positively with the triceps skinfold Z score and arm muscle area Z score between four and five.

**Conclusions:**

Among studied infants and prepubertal children, serum IGF-1 was significantly higher in girls than boys and was considerably lower in children with overweight or obesity. Breastfeeding showed no association with IGF-1 levels.

**What’s Known on This Subject**:

- Children’s growth potential is not solely determined genetically; other factors, such as nutrition, hormonal regulation, and psychosocial factors, influence the final children’s growth.
- Good nutritional status is essential for regulating growth hormone and insulin-like growth factor-1 (IGF-1), which is crucial in growth regulation.

**What This Study Adds**:

- Girls had significantly higher IGF-1 levels than boys.
- Children with obesity had significantly lower IGF-1 than other children.
- IGF-1 levels were comparable between healthy and children with abnormal nutritional status.
- Breastfeeding showed no association with IGF-1.

## Introduction

Children’s growth potential is not solely determined genetically; other factors, such as nutrition, hormonal regulation, and psychosocial factors, influence the final children’s growth patterns [1].

Nutrition is considered a key component of growth and development in young children. Adequate nutrition is crucial for children to grow healthy and develop excellent intellectual skills, emotions, and behavior [2].

Malnutrition among children is a significant public health problem worldwide. It includes under-nutrition (underweight, wasting, or stunting) and over-nutrition (overweight and obesity). Children under five are particularly prone to macro- and micronutrient deficiencies due to their rapid growth and subsequent high nutritional needs [3].

A more complex interplay exists between factors, mainly nutrition, and hormonal factors. Good nutritional status is essential for regulating the axis of growth hormone (GH) and insulin-like growth factor-1 (IGF-1), which is crucial in growth regulation. GH stimulates the transcription of IGF-1 in the liver, subsequently increasing its levels and enhancing its growth-promoting effects [4].

IGF-1 is a polypeptide hormone similar in molecular structure to insulin. It is crucial for prenatal and postnatal growth. It is produced mainly in the liver and mediates various growth-promoting effects of GH in peripheral tissues [5]. IGF-1 has multiple anabolic actions, such as peripheral glucose uptake, increased protein synthesis, amino acid uptake by the skeletal muscles, and reduced proteolysis [1]. Low levels of IGF-1 can inhibit linear growth, affecting weight and height [6].

Although nutritional status is the main regulatory factor for producing IGF-1, other factors also affect its levels, such as age, sex, ethnicity, acute infections, chronic illness, and gene polymorphisms. Therefore, these confounding factors should be considered when evaluating the serum IGF-1 concentration [7].

Under-nutrition strongly affects IGF-1 and can affect the GH/IGF-1 axis at multiple levels, including decreasing expression of GH receptors and IGF-1 transcription in the liver, accelerating IGF-1 decomposition, and reducing the bioavailability of serum IGF-1. These effects could be reversible after a nutritional intervention [4].

There is disagreement regarding the effect of over-nutrition on IGF-I levels; whether there are positive or negative correlations with body mass index (BMI) remains unclear [8].

Although some have advised that measuring the serum level of IGF-1 can complement the growth status assessment and predict growth response [6], its use as an indicator of nutritional status in young children has yet to be thoroughly studied. Therefore, our present study aimed to estimate IGF-1 levels in a group of apparently healthy young children and compare the levels among children with different nutritional statuses, i.e., healthy nutritional status and malnutrition.

## Methodology

Our cross-sectional study included 252 infants and young prepubertal children with a mean age of 3.7 ± 1.3 years (1.1–6.6) for IGF-1 level estimation. This work is a sub-study of a screening project for screening of anemia and abnormal nutritional status in young children, which was conducted at Helwan University Hospital between November 2021 and November 2022 [9]. The research ethics committee of the faculty of medicine at Helwan University provided the initial project approval (Serial: 11-2021) and an additional approval for IGF-1 testing during the project amendment (Serial: 10-2022 R). Children enrolled in the project after obtaining broad consent for participation and publication from one of their parents.

The project included apparently healthy Egyptian prepubertal children of both genders aged between one and seven years. Children with acute illness, chronic diseases, or chronic anemia were excluded.

Children with sufficient blood samples for IGF-1 testing were enrolled in the current work. Our current analysis used the following data: demographic data, breastfeeding history, and anthropometric measurements. We measured weight (Kg) using a scale, and height (cm) was assessed using a stadiometer. To calculate BMI, we divided the weight by height in square meters. The Z score of the measures taken was evaluated by an online growth calculator [10]. We chose the WHO standards for those below two years and the CDC growth references for those aged two or more as per the American Academy of Pediatrics.

Besides, we measured the mid-upper arm circumference (MUAC) in cm, midway between the olecranon process (tip of the elbow) and the acromial process of the left arm using flexible tape [11]. MUAC less than 12.5 cm in under five, and less than 14.5 cm in those aged between 5 and 9, was considered acute malnutrition [12]. In addition, we used a Slim Guide skinfold caliper to measure the triceps skinfold thickness (TSF) in mm. TSF was measured at the same point for MUAC, with the arm hanging freely at the side and the caliper applied vertically [13]. The Z score of MUAC, TSF, and percentiles of arm muscle area and arm fat area measurements were calculated using the same online growth calculator [10].

### Definitions of abnormal nutritional status detected with anthropometric measures

- Under-nutrition: when values are below − 2 Z score [13, 14]:
  - *Underweight: low weight-for-age.*
  - *Wasting (acute malnutrition): low weight-for-height & thinness: low body mass index.*
  - *Stunting: low height-for-age and weight-for-age.*
  - *Short stature: low height-for-age with normal or high weight-for-age.*

*Children with Z score below − 2 were further classified into moderate cases between − 2 and − 3 or severe when below − 3.*

- Over-nutrition: using *BMI* Z score [13, 14]:
  - *Overweight: when the Z score was between + 2 and + 3 in under 5 or between + 1 and + 2 in children above five years.*
  - *Obesity: when the Z score was above + 3 in children under five and above + 2 in children above five years.*

Blood samples were withdrawn in the morning in a plain tube, promptly centrifuged, and stored at − 20 C until analysis. After collecting all samples, IGF-1 was measured using an enzyme-linked immunosorbent assay (ELISA kits) according to manufacturer instructions. Its Catalog Number was DRG^®^ IGF-1 600 ELISA (EIA-4140), DRG International, Inc., USA. The equipment used was the Stat fax Eliza reader 3300 from Gamma Trade Company. The recommended IGF-1 Kit reference range for prepubertal children aged between 3 and 8 years was 20–250 ng/ml; for younger children, it was under 50 ng/ml. The manufacturing company recommended individualizing normal and abnormal values for each laboratory.

### Statistical analysis

Our sample size was reached when all the available kits were consumed. Data was analyzed using SPSS 26 for Windows (Statistical Package for Social Science).

Normally distributed numerical data was presented as mean (± SD), while median (Interquartile range) was used for non-normally distributed data. Frequency (percentage) was used for qualitative data presentation. The student T-test was used to compare two normally distributed data. ANOVA test was used to test IGF-1 among children with different BMI categories. We used the Pearson correlation coefficient to test linear associations between IGF-1 values and different variables. P-value <0.05 was considered statistically significant.

## Results

Our cross-sectional study included 252 healthy young children who participated in a screening project for anemia and nutritional status. Table 1 shows the study group’s demographic data, anthropometric measures, and IGF-1 values. Our participants had a mean age of 3.7 ± 1.3 years (1.1–6.6), with equal gender distribution. Breastfed children represented 86.4% of the study group; among them, 181 (71.8%) breastfed exclusively for a mean duration of 6 ± 1.7 months [4–12], while 37 (14.7%) received formula alone or as supplementation to breastfeeding. In our study group, 83 (32.9%) had a single or multiple abnormal anthropometric measures; 18 (7.1%) were underweight, 18 (7.1%) had short stature, 16 (6.3%) had wasting, and 51 (20.2%) children had a high BMI. Even though about one-fifth of our group had a high BMI, only a small percentage was defined as overweight and obese (4.8%), while the remaining were labeled as having a possible risk of being overweight. None of the study groups had a low MUAC for age.

**Table 1:** Demographic data, Anthropometric Measures, and insulin-like growth factor-1 values of the study group (*N* = 252)

Variable	Value
**Age in years**; mean ± SD [Min-Max]	3.7 ± 1.3 [1.1–6.6]
**Gender**; N (%)	
• *Male*	126 (50%)
• *Female*	126 (50%)
**Infant feeding**; N (%)	
• *Breastfeeding*	218 (86.5%)
• *Never breastfed*	34 (13.5%)
**Anthropometric Measures**; N (%)	
• *Normal*	169 (67.1%)
• *Abnormal*	83 (32.9%)
**Weight**:	
• In Kg; mean ± SD [Min-Max]	15.5 ± 3.7 [6–34.5]
• Z score; median (IQR):	0.2 (-0.9–0.6)
○ **Low weight Z score “Underweight”**; N (%)	**18 (7.1)**
- *Moderate underweight*	12 (4.8)
- *Severe underweight*	6 (2.4)
**Length/Height**:	
• In cm; mean ± SD [Min-Max]	97 ± 10.5 [74–123.5]
• Z score; median (IQR)	-0.5 (-1.1–0.2)
○ **Low Length/Height Z score “Short stature”;** N (%)	**18 (7.1)**
▪ *Short stature; short with normal weight*	*10 (3.97)*
▪ *Stunting (short with underweight)*:	*8 (3.17)*
- *Moderate stunting*	*6 (2.4)*
- *Severe stunting*	*2 (0.8)*
**Weight for length/Body Mass Index in Kg/m**^**2**^:	
• Mean ± SD [Min-Max]	16.3 ± 2 [9.1–23.3]
• Z score; median (IQR)	0.3 (-0.5–1.1)
**-Abnormal Weight for length / BMI Z score**:	
○ **Low “Wasting” or Acute malnutrition;** N (%)	**16 (6.3)**
- *Moderate wasting*	*8 (3.17)*
- *Severe wasting*	*8 (3.17)*
○ **High**; N (%)	**51 (20.2)**
- *Possible risk of overweight in below 5 years*	*39 (15.5)*
- *Overweight*	*9 (3.6)*
- *Obese*	*3 (1.2)*
**Mid-upper arm circumference in cm;** mean ± SD (*N* = 248)	16.8 ± 1.8
• *Z score; median (IQR)*	0.5 (-0.4–1.3)
**Triceps skin fold in mm;** mean ± SD (*N* = 247)	18.7 ± 4.1
• *Z score; median (IQR)*	3.2 (2.4–4.1)
**Arm muscle area;***median (IQR* (*N* = 247)	-2.7 (-3.8 - -1.8)
• *Percentile; median (IQR)*	0.4 (0–3.9)
**Arm fat area;***median (IQR)* (*N* = 247)	1.8 (1.3–2.2)
• *Percentile; median (IQR)*	96.6 (91–98.5)
**Insulin-like growth factor-1 in ng/ml**; mean ± SD [Min-Max]	170.3 ± 42.9 [35–300]

IQR: interquartile range, Min-Max: minimum-maximum, N (%): number (percent), SD: standard deviation

All children lived in an urban city except for one (0.4%) from a rural area. Consanguinity was positive in 52 (20.6%) children. No significant difference was observed between IGF-1 levels between those who breastfed and those who never breastfed (170.5 ± 44.3 vs. 168.7 ± 33.8; P 0.8).

No significant difference was observed between children with normal and abnormal nutritional status, Table 2. Infants and children with overweight or obesity had significantly lower IGF-1 levels than other BMI categories. No significant difference was observed regarding IGF-1 values between cases with moderate and severe underweight, wasting, or short stature. No significant linear associations were observed between IGF-1 levels and age, duration of exclusive breastfeeding, the anthropometric measures obtained or their Z scores (data not presented).

**Table 2:** Insulin-like growth factor-1 in children with normal versus those with abnormal anthropometric measures (*N* = 252)

Normal vs. Abnormal	Insulin-like growth factor-1 Mean ± SD	*P* value
**Anthropometric Measures**:		0.4
• *Normal (N = 169)*	168.8 ± 40.8	
• *Abnormal (N = 83)*	173.9 ± 47.2	
**Weight Z score**:		0.8
• *Normal (N = 169)*	168.8 ± 40.8	
• *Underweight* (*N* = 18)	165.6 ± 47.7	
**Length/Height Z score**:		0.7
• *Normal (N = 169)*	168.8 ± 40.8	
• *Short stature* (*N* = 18)	172.5 ± 46.9	
**-Normal versus Stunting “short with underweight”**:		0.9
• *Normal (N = 169)*	168.8 ± 40.8	
• *Stunting (N = 8)*	170.9 ± 28.7	
**-Normal versus Short stature without underweight**:		0.7
• *Normal (N = 169)*	168.8 ± 40.8	
• *Short stature (N* = 10)	173.8 ± 59.2	
**Weight for length/Body Mass Index Z score**:		0.3
• *Normal (N = 169)*	168.8 ± 40.8	
• *Wasting (Acute malnutrition); (N = 16)*	158.5 ± 50.7	
**Weight for length/Body Mass Index Z score**:		0.05
• *Normal or borderline (N = 240)*	171.4 ± 42.9	
• *Over/obese (N = 12)*	146.8 ± 38.8	
**Weight for length/Body Mass Index Z score**:		0.04*
• *Normal or low (N = 201)*	168.8 ± 42.2	
• *Possible risk of overweight (N = 39)*	185 ± 44.5	
• *Overweight (N = 9)*	147.1 ± 43.2	
• *Obese (N = 3)*	145 ± 28.7	

N: number, SD: standard deviation. *P value is significant at < 0.05

In our study group, girls had significantly higher serum IGF-1 levels than boys. Also, girls with abnormal anthropometric measures, particularly those with short stature and those with overweight or obesity, had substantially higher IGF-1 levels than boys with similar nutritional status (Table 3). Testing linear association in boys and girls showed no significant correlations between IGF-1 values and obtained anthropometric measures and their Z scores (Data not presented).

**Table 3:** Comparison of insulin-like growth factor-1 between boys and girls in our study group

	Boys	Girls	*P* value
	Mean ± SD (N)	Mean ± SD (N)	
Insulin-like growth factor-1 in ng/ml	161.8 ± 44.2 (*N* = 126)	178.8 ± 40.2 (*N* = 126)	< 0.001*
Normal Anthropometry; (*N* = 169)	163.5 ± 41 (*N* = 92)	175.1 ± 39.8 (*N* = 77)	0.07
Abnormal Anthropometry; (*N* = 83)	157.1 ± 52.1 (*N* = 34)	184.5 ± 40.4 (*N* = 49)	< 0.001*
Underweight; (*N* = 18)	167.8 ± 67.7 (*N* = 8)	163.8 ± 27.1 (*N* = 10)	0.9
Short stature cases; (*N* = 18)	141.8 ± 35.5 (*N* = 8)	197.1 ± 40.8 (*N* = 10)	< 0.001*
*- Stunted children;* (*N* = 8)	162.5 ± 38.5 (*N* = 4)	179.3 ± 16 (*N* = 4)	0.4
*- Short stature with normal weight;* (*N* = 10)	121 ± 17.5 (*N* = 4)	209 ± 49.1 (*N* = 6)	0.01*
Wasting (Acute malnutrition); *(N = 16)*	162.1 ± 72.5 (*N* = 7)	155.7 ± 29.2 (*N* = 9)	0.8
Overweight & Obese; *(N = 12)*	133.9 ± 41.6 (*N* = 8)	172.5 ± 12.6 (*N* = 4)	0.04*

N: number, SD: standard deviation. *P value is significant at < 0.05

We categorized our participants into five groups according to their ages and compared the IGF-1 levels between boys and girls. Among all age categories, girls had higher IGF-1 levels than age-matched boys, which reached statistical significance among those aged between 4 and 5 (Table 4). IGF-1 levels showed no significant difference among children in different age groups.

**Table 4:** Comparison of insulin-like Growth Factor-1 values between different age groups in our Studied Children (*N* = 252)

Age group	One to 2 years		Between 2 & 3 years		Between 3 & 4 years		Between 4 & 5 years		Above 5 years	
N (% of total)	12 (4.8)		75 (29.8)		62 (24.6)		49 (19.4)		54 (21.4)	
IGF-1 in ng/ml: mean ± SD [Min-Max]	166.9 ± 33.8 [120–220]		171.8 ± 45.3 [35–250]		165.98 ± 42.8 [35–250]		172.5 ± 48 [56–300]		168.7 ± 42.4 [65–260]	
*Gender*	*Boys*	*Girls*	*Boys*	*Girls*	*Boys*	*Girls*	*Boys*	*Girls*	*Boys*	*Girls*
- N (%)	3 (25)	9 (75)	39 (52)	36 (48)	38 (61.3)	24 (38.7)	20 (40.8)	29 (59.2)	26 (48.1)	28 (51.9)
- Mean ± SD	156 ± 55.4	170.6 ± 27.2	168 ± 40.3	180.4 ± 43.4	160.8 ± 44.7	174.2 ± 39.3	152.8 ± 46.7	186.1 ± 44.7	161.3 ± 47.9	175.6 ±36
- *P value*	*0.7*		*0.2*		*0.2*		0.02*		*0.2*	

IGF-1: Insulin-Like Growth Factor-1, Min-Max: minimum-maximum, N (%): number (percent), SD: standard deviation. *P value is significant at < 0.05

Testing the IGF-1 levels and anthropometric measurements at different age groups showed that IGF-1 levels positively correlated with arm muscle area Z score in infants and toddlers. Meanwhile, IGF-1 levels correlated positively with Z scores of weight and BMI in children between three and four years. Between four and five years, IGF-1 levels correlated positively with the triceps skinfold’s Z score and the Z score of the arm muscle area (Table 5).

**Table 5:** Correlations between insulin-like growth factor-1 values and anthropometric measures in different age groups in our Studied Children (*N* = 252)

IGF	One to 2 years (*N* = 12)		Between 2 & 3 years (*N* = 75)		Between 3 & 4 years (*N* = 62)		Between 4 & 5 years (*N* = 49)		Above 5 years (*N* = 54)	
	*r*	*P* value	*r*	*P* value	*r*	*P* value	*r*	*P* value	*r*	*P* value
Anthropometry										
Weight in Kg	-0.08	0.5	-0.08	0.5	0.2	0.05	0.1	0.5	-0.04	0.8
Weight Z score	-0.04	0.8	-0.04	0.8	0.3	0.03*	0.08	0.6	-0.08	0.6
Height in cm	-0.1	0.3	-0.1	0.3	0.08	0.5	0.04	0.8	0.09	0.5
Height Z score	-0.07	0.6	-0.07	0.6	0.1	0.4	-0.03	0.9	0.03	0.8
WFL/BMI	-0.008	0.9	-0.008	0.9	0.2	0.1	0.1	0.5	-01	0.3
WFL/BMI Z score	0.001	0.9	0.001	0.9	0.3	0.03*	0.2	0.2	-0.1	0.4
MUAC	0.04	0.7	0.04	0.7	0.04	0.8	-0.2	0.2	-0.04	0.8
MUAC Z score	0.005	0.9	0.005	0.9	0.05	0.7	-0.03	0.8	-0.2	0.2
Triceps skinfold thickness	-0.2	0.1	-0.2	0.1	0.07	0.6	0.3	0.06	-0.06	0.7
Triceps skinfold Z score	-0.2	0.2	-0.2	0.2	0.04	0.7	0.3	0.02*	-0.2	0.2
Arm muscle area Z score	0.2	0.04*	0.2	0.04*	0.02	0.9	0.3	0.03*	-0.03	0.8
Arm muscle area percentiles	0.2	0.1	0.2	0.1	-0.08	0.6	-0.2	0.2	0.06	0.7
Arm fat area Z score	-0.1	0.3	-0.1	0.3	0.06	0.6	0.2	0.2	-0.1	0.4
Arm fat area percentiles	-0.2	0.1	-0.2	0.1	0.08	0.6	0.2	0.2	-0.1	0.3

MUAC: Mid-upper arm circumference, N: number, r: Pearson Correlation, WFL/BMI: weight for length/body mass index. *P value is significant at < 0.05

## Discussion

Our cross-sectional study included 252 healthy young prepubertal children with a mean age of 3.7 ± 1.3 years (1.1–6.6) and equal gender distribution. We aimed to evaluate IGF-1 levels and compare levels among participants with different nutritional statuses.

Our findings showed significantly higher levels of serum IGF-1 in girls than in boys. This gender discrimination was maintained when we compared girls and boys with abnormal anthropometric measures. This observation agrees with studies of different pediatric populations. Similar findings were reported by Yüksel et al. [15], who studied healthy infants and children below six years of age, with a boy-to-girl ratio of 1:1.2. All participants had weight and height within the normal range according to national standards. They reported higher serum IGF-1 in girls than in aged-comparable boys. Moreover, Kjaer et al. [16] observed higher serum IGF-1 levels among infants with moderately acute malnutrition in girls than boys. They included 1546 infants aged 6–23 months, whose boy-to-girl ratio was 1:1.2.

Our cases with abnormal anthropometric measures (32.9%) had a comparable IGF-1 level to children with average measures. In addition, children with abnormal nutritional status, i.e., short stature, underweight, or wasting, had a comparable IGF-1 to children with average measures. Our findings disagree with studies concerned with malnourished infants and children, where infants and prepubertal children who are underweight [17] or stunted (with average weight or underweight) [6; 18] had significantly lower IGF-1 compared to normal comparable controls.

Among our participants, most participants breastfed at infancy (87%), and 71.8% exclusively for at least four months. The rest of our group received supplementary formula. No significant difference was observed between serum IGF-1 levels in those who breastfed and those who did not. Studies concerning breastfeeding and IGF-1 level yield controversial results. Madsen and colleagues [19] reported lower IGF-1 levels in breastfed children, while Kjaer and coworkers [16] observed lower serum IGF-1 in malnourished infants who never breastfed. The timing of the assessment in relation to breastfeeding may explain this difference. In addition, infants who received formula had higher concentrations of IGF-1, as formulas contain higher protein concentrations than breast milk [20].

Among our cohort, children with obesity had a lower IGF-1 level than the rest of the group but did not reach statistical significance. When we used the ANOVA test among different BMI categories, children with overweight and obesity (4.8%) had significantly lower IGF-1 values than other categories. Pediatric reports have a controversy concerning the association between IGF-1 values and obesity. Previous studies observed similarly lower levels of IGF-1 in children with obesity [21]. Other studies reported higher [17, 22, 23] or comparable [24] IGF-1 values in children with high BMI compared to lean children. Different sample sizes, ages, stages of puberty, and definitions used for obesity are among the factors that explain the observed difference in the relationship between serum IGF-1 and BMI. In addition, the small number of overweight or obese participants, 12 out of 252, may explain our findings, with an expected change in the significance level (P: 0.04) with an increased number of the overweight group in future comparative analysis.

Most of our participants had average anthropometric measures; a few had short stature or stunting. Our cohort results showed no significant linear associations between IGF-1 levels and age or the Z score of the growth measures obtained. Similar findings were observed when we tested girls and boys separately. Studies concerned with participants who were stunted or short yielded different results. Wen et al. [25] reported positive correlations between IGF-1 and age, weight, BMI, and standard deviation scores of weights and BMI (*P* < 0.001). They studied 333 children with a height below the 10th percentile, aged between 5 and 14 years, with a 1:1.8 boy-to-girl ratio. Moreover, Abdou et al. [6] reported positive correlations between IGF-1 and age, weight, and height in their stunted children (with average or underweight). However, IGF-1 showed a significant positive correlation with height for age Z score in stunted underweight children only.

Our findings showed significant correlations between IGF-1 and anthropometric measures when we categorized the patients by age. On revising available literature, variable results of linear association have been reported, depending on the age and nutritional status of the study group. In infants under two years with moderate acute malnutrition, the Z score for weight, length, and mid-upper arm circumference (*P* ≤ 0.001) correlated positively with serum IGF-1. Also, fat-free mass showed positive correlations with IGF-1, which disappeared after length adjustment [16]. In preschools with standard anthropometry, serum IGF-1 levels correlated positively with BMI [15]. In children with stunting and wasting, aged between 6 and 9 years, serum IGF-1 values were associated with fat-free mass [26].

The main limitation of our study included its cross-sectional nature, with limited potential to demonstrate cause-effect relationships as expected between IGF-1 and growth rate. Also, growth hormone assessment was not available for testing children with short stature. Future longitudinal studies are essential to highlight the relation between serum IGF-1 levels and growth in children with normal and abnormal nutritional status.

A strength of the study is its considerable sample size, the variable nutritional status of the studied children, and the high sensitivity and specificity of the used IGF-1 kit. It is, besides, testing IGF-1 in apparently healthy infants and children.

## Conclusion

Among our studied infants and prepubertal children, girls had significantly higher levels of IGF-1 than boys. Children with obesity had significantly lower IGF-1 values than those with other BMI categories. Serum IGF-1 levels were comparable between children with normal versus those with abnormal growth measures. No significant correlations were observed between breastfeeding and IGF-1 levels.

## Data Availability

Availability of data and material: The datasets used and/or analyzed during the current study are available from the corresponding author upon reasonable request.
